# Genetic Variations in CYP19A1 and SLCO1B1 Genes and Their Association with Endometrial Cancer Risk in the Taiwanese Population: A Case–Control Study

**DOI:** 10.3390/ijms26062461

**Published:** 2025-03-10

**Authors:** Yu Wang, Yu-Ru Wu, Tzu-Hung Hsiao, I-Chieh Chen, Hsiao-Fan Kung

**Affiliations:** 1 Division of Reproductive Endocrinology and Infertility, Department of Obstetrics, Gynecology and Women’s Health, Taichung Veterans General Hospital, Taichung 40705, Taiwan; 2 Department of Medical Research, Taichung Veterans General Hospital, Taichung 40705, Taiwan; 3 Department of Public Health, Fu Jen Catholic University, New Taipei City 24205, Taiwan; 4 Institute of Genomics and Bioinformatics, National Chung Hsing University, Taichung 40227, Taiwan

**Keywords:** gene, endometrial cancer, genome-wide association study (GWAS)

## Abstract

Endometrial cancer is the most common gynecologic malignancy in developed countries, and its incidence is rising globally. Genetic predisposition plays a significant role in modulating risk, particularly in Asian populations. In Taiwan, the burden of endometrial cancer has increased, highlighting the need to gain a better understanding of the genetic loci associated with this disease. This retrospective case–control study included 373 endometrial cancer patients and 3730 controls from the Taiwan Precision Medicine Initiative. Genotype data were obtained using the TWB 2.0 SNP chip. Statistical analyses were conducted using PLINK and SPSS, with logistic regression models assessing the associations between genetic variants and endometrial cancer risk. In this study, we identified two SNPs, rs17601876 in CYP19A1 and rs2900478 in SLCO1B1, that were associated with endometrial cancer. The AG/GG genotypes of rs17601876 showed a protective effect (OR = 0.743, *p* = 0.006), while the TA/AA genotypes of rs2900478 exhibited a nonsignificant trend toward an increased risk. Higher BMI, LDL, triglyceride, total cholesterol, and HbA1c, as well as lower HDL, were strongly associated with greater risk. Our findings demonstrated a protective role of rs17601876 in CYP19A1 and further showed its potential impact on estrogen biosynthesis. Genetic factors involved in endometrial cancer risk are an important issue. Further functional studies are needed to validate the present findings.

## 1. Introduction

Endometrial cancer is the most common gynecologic malignancy in developed countries, and its incidence is on the rise globally. Previously, endometrial cancer was considered more common in Western than in Asian countries. In Taiwan, the burden of this disease has grown in recent years, making it a critical public health concern [1]. In 2021, uterine corpus cancer was ranked as the fifth most common cancer in women in Taiwan, accounting for 17 cases per 100 thousand population. Globally, the latest data from Bray et al. [2] indicate that corpus uteri cancer is the sixth most common cancer among females. The study also reported that countries with a high or very high Human Development Index (HDI) have an incidence rate more than three times higher than those with a lower HDI.

The etiology of endometrial cancer is multifactorial, involving hormonal, environmental, and lifestyle factors. However, growing evidence has revealed the importance of genetic predisposition in modulating an individual’s risk of developing the disease. For instance, women with Lynch syndrome or a family history of endometrial cancer have a two-fold higher risk of developing the disease [3]. Additionally, mutations in mismatch repair genes such as MLH1, MSH2, MSH6, and PMS2 are now known to be associated with approximately 3% of endometrial cancer cases [4]. Beyond these well-established mutations, other genetic variations in the general population may also contribute to endometrial cancer susceptibility.

The genome-wide association study (GWAS) is instrumental in identifying genetic variants associated with cancers. Such studies have provided insights into specific polymorphisms that influence endometrial cancer risk. Among these, certain genetic variants showed potential protective effects, despite unclear underlying mechanisms. The first endometrial cancer GWAS was conducted in 2011. A single risk region of susceptibility was identified close to HNF1B on chromosome 17q [5]. The next large GWAS on >12,000 endometrial cancer cases was conducted by the Endometrial Cancer Association Consortium [6] and it identified nine genome-wide loci. A further review study included more candidate susceptibility genes using functional analyses to try to generate endometrial genetic risk scores [7,8].

One of the most common concerns among patients diagnosed with endometrial cancer is whether their daughters or family members may inherit the same disease. Despite the availability of guidelines for endometrial cancer screening and treatment, as well as genetic testing and risk assessment, genetic testing and counseling remain expensive in many countries [9,10]. Additionally, the availability of trained healthcare professionals in this field is limited. To achieve cost-effective and clinically meaningful results, it is crucial to tailor genetic data to specific populations and ethnic groups, ensuring that risk assessments and treatment strategies are optimized for different demographics. Research in genetic susceptibility can help address these concerns by identifying hereditary risk factors. For example, a previous study by Wang et al. [8] identified novel genetic loci associated with testosterone regulation, a factor not previously linked to endometrial cancer. Such findings highlight new opportunities for targeted therapy development and potential preventive strategies.

With the availability of population-specific genetic data, we now have an opportunity to apply these findings to clinical practice. Integrating genetic insights into patient care could enhance risk prediction, guide personalized treatment strategies, and potentially pave the way for new therapeutic approaches in endometrial cancer management.

In this study, we aimed to analyze Taiwanese genetic data to investigate whether any genetic loci are associated with an altered risk of developing endometrial cancer in the local population. Given the importance of precision medicine, we recommend that genetic risk assessment should be tailored to local population data, as this approach is crucial not only for treatment but also for disease diagnosis and prevention.

## 2. Results

A total of 373 endometrial cancer patients and 3730 controls were included in this study. The ages of the two groups were matched with a ratio of patients to controls of 1:10. We found no significant inter-group differences in the prevalence of hypertension, diabetes mellitus, or chronic kidney disease. However, the endometrial cancer patients had a lower prevalence of hyperlipidemia and a higher BMI. Although the endometrial cancer patients had a lower reported diagnosis of hyperlipidemia, they exhibited higher levels of LDL, triglycerides, total cholesterol, and HbA1c, while their HDL levels were lower compared to the control group. This discrepancy may suggest underdiagnosis of hyperlipidemia in the endometrial cancer group or differences in lipid metabolism between the groups (Table 1). The data are presented as mean (SD) for the continuous variables while the categorical variables are presented as n/t (%), where n represents the number of individuals in the category and t is the total number of individuals who provided data for that variable. To test the difference between the cancer and control groups, the two-sample *t*-test for continuous factors or the chi-square test for dichotomous factors were used in the analysis.

Regarding disease characteristics, most endometrial cancer cases were diagnosed at an early stage and received surgical treatment, with a 5-year survival rate > 99%. Among the patients with available records, we found a low prevalence of alcohol consumption, betel nut chewing, and smoking habit (Table 2).

Based on the GWAS findings reported by Wang et al. [7], we identified 48 SNPs. Of these, 17 SNPs were available in our database (Supplementary Table S1). Using the chi-square test to compare carrier proportions between the case and control groups, we found that rs2900478 in SLCO1B1 and rs17601876 in CYP19A2 displayed significantly different distributions, indicating a potential association with endometrial cancer. The genotype frequencies for these variants are presented in Table 3. The basic information of SNPs in SLCO1B1 and CYP19A1, including gene position, allele, SNP function annotation, and genotype frequency distribution, are shown in Supplementary Tables S2 and S3.

Univariable logistic regression was performed, which revealed the associations of demographic factors, comorbidities, blood biomarkers, and SNPs (rs2900478 and rs17601876) with the risk of endometrial cancer, as shown in Table 4. Each factor was analyzed individually without adjusting for the other variables. The univariable logistic regression analysis indicated that a BMI over 24, as well as higher LDL, triglyceride, total cholesterol, and HbA1c, but lower HDL, were associated with a higher risk of endometrial cancer. Among the genetic variants that were analyzed, TA and AA genotypes of rs2900478 in SLCO1B1 exhibited a trend toward an increased risk of endometrial cancer (OR = 1.196, 95% CI: 0.9742–1.4694, *p* = 0.087), but the association did not reach statistical significance. In contrast, the AG and GG genotypes of rs17601876 in CYP19A2 were significantly associated with a reduced risk of endometrial cancer (OR = 0.743, 95% CI: 0.6012–0.9175, *p* = 0.006), when compared with their respective reference genotypes. The Stratified analysis of rs2900478 and rs17601876 age is also shown in Supplementary Table S4.

The multivariable logistic regression analysis revealed the effects of BMI ≥ 24, rs17601876, and hyperlipidemia on the endometrial cancer risk (Table 5). The AG/GG genotypes of rs17601876 were linked to a lower risk of endometrial cancer (OR = 0.832, *p* = 0.028 in Model 2), indicating a protective effect. In contrast, rs2900478 with TA and AA genotypes did not show a significant trend in this analysis. Hyperlipidemia and BMI emerged as two factors that were associated with endometrial cancer. No such significant association was found with other factors such as age, diabetes mellitus, chronic kidney disease, and hypertension.

**Figure 1:**
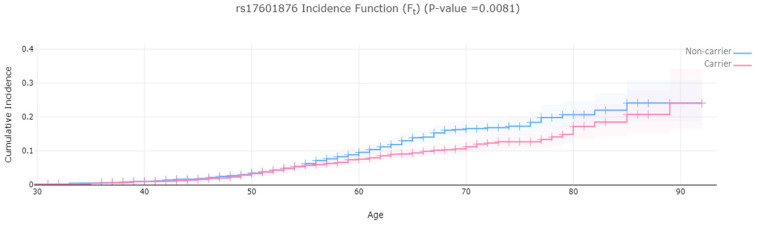
The incidence function of carrier and non-carrier of rs17601876.

**Figure 2:**
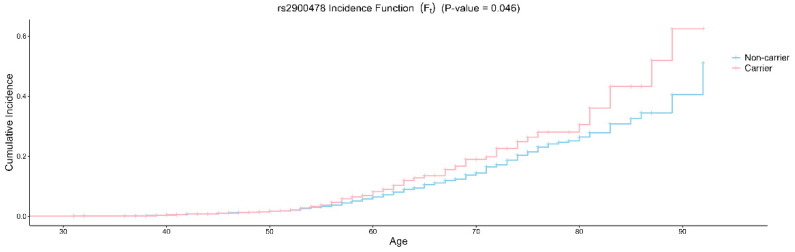
The incidence function of carrier and non-carrier of rs2900478.

Figure 1 illustrates the cumulative incidence function for rs17601876 carrier status and shows the probability of developing endometrial cancer over time. Two survival curves are plotted: the blue line represents non-carriers of rs17601876, while the pink line represents carriers. The x-axis represents age (ranging from 30 to 95). The y-axis represents the cumulative incidence (F_t_), indicating the increasing probability of disease occurrence as age progresses. The *p*-value (0.0081) suggests a statistically significant difference in cumulative incidence between carriers and non-carriers. Figure 1 supports the protective role of rs17601876 and is consistent with the regression results. Figure 2 illustrates the cumulative incidence function for rs2900478 carrier status and shows the probability of developing endometrial cancer over time. The *p*-value (0.046) suggests a statistically significant difference in cumulative incidence between carriers and non-carriers. Figure 2 shows that rs2900478 increases the risk of endometrial cancer.

## 3. Discussion

Endometrial cancer is a complex disease influenced by an interplay of genetic, environmental, and hormonal factors. In this study, we determined the association of specific genetic loci with endometrial cancer risk in the Taiwanese population. Our findings support the growing evidence that genetic predisposition plays a critical role in modulating the risk of endometrial cancer. In particular, our results highlight the significance of the SNPs rs17601876 and rs2900478 in influencing susceptibility to endometrial cancer.

The growing incidence of endometrial cancer in Taiwan mirrors global trends and contradicts the conventional belief that Asian populations have a risk lower than Western populations [1]. This rise in endometrial cancer incidence underscores the importance of identifying specific risk factors, like genetic predispositions, for targeted prevention and treatment strategy.

Our results showed that the AG and GG genotypes of rs17601876 were associated with a reduced risk of endometrial cancer, consistent with findings in previous genome-wide association studies (GWAS) [7,8]. According to data from the National Center for Biotechnology Information (NCBI) [11], the overall carrier frequency of this variant is 46.67% in the general population and 34.3% in Asians. In our study, the carrier frequency was 47.18% in the endometrial cancer group and 54.79% in the control group. Compared with NCBI data, our findings indicate a relatively similar prevalence in the endometrial cancer group but a higher prevalence in the control group. This difference may contribute to the lower prevalence of endometrial cancer in the Taiwanese population. Notably, a search of the literature revealed that only four studies have cited rs17601876 to date. The protective role of rs17601876 in the CYP19A1 gene underscores the critical role of aromatase in modulating the estrogen level, which is a key driver in the development and progression of hormone-dependent cancers such as endometrial cancer and breast cancer [12]. This gene controls the final step in estrogen biosynthesis and converts androgens to estrogen. Androgens are important for the synthesis of estrone and 17β-estradiol, especially after menopause. CYP19A1 shows the highest expression in adipose tissue. In adipose tissue, CYP19A1 produces estrogens, which are released into blood circulation [13]. The CYP19A1 gene encodes aromatase, an enzyme that is crucial for a variety of biological processes, including reproductive health and the progression of cancers that are influenced by hormones [14]. Elevated estrogen levels have been implicated in the pathogenesis of endometrial cancer. A pooled analysis by Setiawan et al. [15] examined two single nucleotide polymorphisms (SNPs), rs749292 and rs727479, in the CYP19A1 gene across 4998 endometrial cancer cases and 8285 controls. The study found that carrying the A allele of rs749292 was associated with a 14% increased risk per allele (OR per allele = 1.14, 95% CI: 1.09–1.21), and the A allele of rs727479 was associated with an 8% increased risk per allele (OR per allele = 1.08, 95% CI: 1.02–1.14). The rs17601876 variant is also associated with other diseases such as stroke, type 2 diabetes mellitus, and bladder cancer, indicating its potential influence on disease risk through the aromatase pathway [16,17,18]. The rs17601876 variant, found to be protective in our cohort, may also influence gene–environment interactions specific to the Taiwanese population. Functional studies in the future exploring rs17601876’s impact on gene expression and protein function in endometrial tissue may provide insights into its protective role. This SNP may influence regulatory pathways involving hormone signaling or DNA repair, but further functional studies are needed to elucidate the exact mechanisms. Moreover, given that CYP19A1 exhibits the highest expression in adipose tissue, individuals with a higher BMI may have increased aromatase activity, leading to greater estrogen production. Chronic elevation of estrogen levels has been implicated in the pathogenesis of endometrial cancer, as persistent estrogen stimulation without adequate progesterone counteraction promotes endometrial proliferation and malignant transformation [19,20,21].

Interestingly, the TA and AA genotypes of rs2900478 were found to be associated with an increased risk in univariable analysis, although this trend was not statistically significant in multivariable models. This discrepancy shows the existence of complex interactions between genetic and non-genetic factors. rs2900478 may still be relevant in larger studies or when analyzed alongside other genetic markers, suggesting the need for further investigation into its role in endometrial cancer. According to data from the NCBI, the carrier frequency of this variant is 15.61% in the general population and 17% in Asians [22]. In our study, the carrier frequency was 23.32% in the endometrial cancer group and 19.02% in the control group. Compared with NCBI data, our findings indicate a higher prevalence in both groups, with an even greater frequency observed in the endometrial cancer cohort. Notably, a literature search revealed that only four studies have cited this SNP to date. The SNP rs2900478 is located within the SLCO1B1 gene, which encodes the hepatic transporter protein OATP1B1. This protein is integral to the hepatic uptake of various compounds, including statins—a widely prescribed class of drugs for reducing low-density lipoprotein cholesterol levels. SLCO1B1 is involved in the hepatic uptake of various endogenous compounds, including steroid hormones, and its variants may influence hormone levels and cancer susceptibility. These findings are consistent with previous studies highlighting the role of hormone-related pathways in endometrial carcinogenesis [23]. Variants in SLCO1B1, such as rs2900478, have been shown to influence the pharmacokinetics and efficacy of statins [24]. Moreover, rs2900478 has been implicated in pathways beyond cholesterol metabolism. Previous studies have associated this SNP with the estrone pathway, suggesting its role in estrogen metabolism and signaling. Dysregulation in this pathway likely contributes to hormone-related cancers, including estrogen-receptor-positive breast tumors [25]. Given the shared hormonal pathways involved in breast and endometrial cancer, it is plausible that rs2900478 may similarly influence endometrial cancer risk through its effects on estrogen biosynthesis or signaling. A study by Gaudet et al. [26] investigated polymorphisms in hormone metabolism pathway genes, including SLCO1B1, and their association with breast cancer risk. However, the study failed to find an association between SLCO1B1 and breast cancer. Functional studies and pathway analyses are needed in the future to clarify its biological impact, particularly in the context of endometrial cancer, where estrogen-driven mechanisms are central to disease development and progression.

A high BMI was found to be strongly associated with an increased risk of endometrial cancer, aligning with the well-documented role of obesity as a major risk factor for this disease [6,27]. Obesity promotes endometrial cancer development through greater estrogen production from adipose tissue, chronic inflammation, and insulin resistance [28]. Obesity is also associated with CYP19A1 as adipose tissue has the highest level of CYP19A1. Patients with a higher BMI likely convert more estrogen from androgens [13].

Contrary to the traditional view of LDL cholesterol as a risk factor for cardiovascular diseases, some studies have observed an inverse relationship between LDL levels and endometrial cancer risk. For instance, a Mendelian randomization analysis indicated that genetically elevated LDL cholesterol levels were associated with a reduced risk of endometrial cancer across all histological subtypes [29]. This association remained significant even after adjusting for BMI, suggesting an independent protective effect of LDL cholesterol against endometrial cancer development. Another study showed serum lipids level to be unrelated to endometrial cancer risk with or without adjusting for BMI [30]. The role of HDL cholesterol in endometrial cancer risk appears to be more complex. While HDL is generally considered protective against cardiovascular diseases, higher genetically predicted HDL cholesterol levels have been associated with an increased risk of non-endometrioid endometrial cancer [29]. The association between total cholesterol levels and endometrial cancer risk remains inconclusive. Some studies have reported no significant relationship between total cholesterol levels and the risk of developing hormonally driven cancers, including endometrial cancer. However, other research has demonstrated a U-shaped relationship between total cholesterol levels and all-cause mortality in cancer patients, suggesting that both low and high cholesterol levels could influence cancer prognosis [31]. However, in our study, we found that higher LDH, higher total cholesterol, and lower HDL were all associated with increased endometrial cancer risk.

A previous study reported that the prevalence of hyperlipidemia in Taiwan, including in both genders, was 29.26% in 2009 and 2010 [32]. Additionally, the study noted a higher prevalence of hypertriglyceridemia in aboriginal areas. This suggests that the control group in our study has a prevalence more comparable to that of the general population. Interestingly, although the endometrial cancer patients exhibited higher levels of LDL, triglycerides, and HbA1c, they had a lower reported diagnosis of hyperlipidemia compared not only to the control group but also to the general population. While we do not have data on income levels or educational attainment for the patient group, socioeconomic factors may play a crucial role in endometrial cancer prevention and treatment accessibility. This discrepancy may be attributed to differences in healthcare access, diagnostic practices, or other underlying factors, which warrant further investigation. A deeper understanding of these disparities could provide valuable insights into metabolic health, healthcare utilization, and preventive strategies in endometrial cancer patients.

The GWAS has greatly advanced our understanding of the genetics underlying endometrial cancer. The identification of susceptibility loci, including rs17601876 and regions near HNF1B, highlights the genetic heterogeneity of the disease [5,6]. Notably, the study by O’Mara et al. identified nine loci associated with endometrial cancer, reflecting the polygenic nature of its etiology [5]. Similarly, research by Cheng et al. identified five new risk loci through GWAS meta-analysis [33]. In our Taiwanese cohort, we observed associations at loci that have been previously reported in other populations, suggesting potential shared genetic risk factors. However, certain loci exhibited different effect sizes or were unique to our study, indicating possible population-specific genetic influences. This aligns with the findings from a study on Chinese patients, which identified distinct genomic profiles in endometrial carcinoma [34].

Our findings add to this body of evidence and demonstrate the importance of population-specific genetic studies, as loci identified in European populations may not fully capture risks in Asian populations. Understanding genetic susceptibility is particularly important in populations with rising cancer incidence, such as the Taiwanese population. Our findings could inform genetic counseling and public health initiatives aimed at reducing the burden of endometrial cancer. One of the most significant advancements in modern medicine is precision medicine, which plays a crucial role, not only in treatment, but also in disease diagnosis and prevention. Genetic variation differs among populations; therefore, conducting appropriate analyses based on local population data is essential. The findings from this study may serve as a foundation for risk assessment when combined with data from other studies. Additionally, this genetic information could contribute to future treatment strategies beyond currently recognized mutations.

There were some limitations in this study, as follows. First, certain detailed clinical and lifestyle information was not recorded. Some factors, such as lifestyle, dietary habits, and hormonal profiles, that could influence endometrial cancer risks were not fully available. Also, while BMI and hyperlipidemia were included as confounders, other potential genetic and environmental interactions were not.

Second, our analysis focused on two specific genetic variants, rs17601876 and rs2900478, so we were not able to rule out other similar genetic loci. The relatively small sample size likely limited the statistical power to detect other genetic variants.

Third, our study cohort was exclusively composed of Taiwanese individuals of Han Chinese ethnicity, which therefore limits the generalizability of our findings to other populations. Genetic susceptibility and environmental exposures vary across ethnic groups, and further studies in diverse populations are necessary to confirm these results.

Lastly, while we observed a protective effect of rs17601876 and a potential risk associated with rs2900478, we did not conduct functional validation. Future research should focus on understanding the biological mechanisms underlying these associations through functional assays and multi-omics analyses. Furthermore, longer-term prospective studies with larger cohorts are needed to validate our findings and evaluate their clinical applicability, such as integrating genetic risk scores into personalized prevention strategies for endometrial cancer.

Future studies are warranted to validate the function of rs17601876, such as its effects on CYP19A1 expression and estrogen biosynthesis in endometrial tissue. Integrating multi-omics data, including transcriptomics and epigenomics, could help elucidate the molecular mechanisms underlying its protective effect. Also, incorporating these findings into genetic risk scores may improve risk stratification and inform personalized prevention strategies.

## 4. Materials and Methods

### 4.1. Participants

In this retrospective case–control study, we analyzed data from the Taiwan Precision Medicine Initiative (TPMI). The cohort comprised Taiwanese participants with Han Chinese ancestry from 16 hospitals across Taiwan, with a significant portion of the cohort consisting of patients from Taichung Veterans General Hospital (TCVGH), a tertiary medical center. A total of 373 patients with endometrial cancer were enrolled. These patients received treatment in TCVGH between June 2019 and May 2021. The control group consisted of female participants from the Taiwan Precision Medicine Initiative who did not have endometrial cancer. The control group serves as a representative sample of the general population and reflects the genetic incidence in the Taiwanese population. For controls, propensity score matching was applied to match the age of the endometrial cancer group with that of the control group, with a ratio of 1:10 to minimize confounding effects.

We identified from the database the following information: genotyping age, history of hypertension, diabetes mellitus, chronic kidney disease, hyperlipidemia, and BMI, and we obtained the following data on tumors: their size, stage, and cell type. The study was conducted in accordance with the Declaration of Helsinki and was approved by the Ethics Committee of Taichung Veterans General Hospital’s Institutional Review Board (IRB no. CE16270B-2 on 25/1/2024). All the available data were obtained from TWB, which collected specimens and information in a complete and standardized procedure to fit researchers’ needs in different fields.

### 4.2. Genotyping and Quality Controls

Genotype data were obtained from participants in the Taiwan Biobank (TWB). A trained medical researcher oversaw the collection of 30 mL blood samples, and DNA extraction was performed using QIAamp DNA blood kits according to the manufacturer’s protocols (Qiagen, Valencia, CA, USA). After assessing the quality of the extracted genomic DNA using previously established methods [35,36], genotyping was carried out using the customized TWB 2.0 SNP chip and the Axiom Genome-Wide Array Plate System (Affymetrix, Santa Clara, CA, USA) at the National Center for Genome Medicine, Academia Sinica, Taiwan. The TWB 2.0 SNP chip, designed specifically for the Han Chinese population in Taiwan, included 653,291 SNPs. Genotype and linkage disequilibrium data were provided by the Ethics and Governance Committee (EGC) of the Taiwan Biobank [37], accessible through TaiwanView (TaiwanView: https://taiwanview.twbiobank.org.tw/index (accessed on 8 March 2025)). PLINK 2.0 alpha software was used for analysis, with quality control steps taken to exclude SNPs failing the Hardy–Weinberg equilibrium (*p* < 1 × 10^−6^), having a minor allele frequency of <0.01, or having a genotyping call rate under 90% [38].

### 4.3. Follow-Up Procedure

Recurrent disease was defined as a tumor detected after 6 months. Routine monthly blood tests were conducted during treatment and at 3-month intervals during follow-up. After the end of therapy and during the first 3 years of follow-up, each patient underwent a bimanual pelvic examination, and a blood test was carried out every 3 months, along with a sonography or abdominal CT every 6 months. From the 3rd year to the 5th year, patients received a bimanual pelvic examination, and follow-up blood tests every 6 months. After 5 years, patients received an annual follow-up with bimanual pelvic examination.

### 4.4. Statistical Analyses

This study employed a case–control design with a 1:10 matching ratio based on age and sex to minimize confounding effects. The Statistical Package for Social Sciences (SPSS version 22.0, Chicago, IL, USA) software for Windows was used to analyze the data. Demographic information was summarized using frequencies and percentages. Parametric data are presented as a means and standard deviations. The chi-square test was applied to evaluate the statistical significance of categorical variables. Univariate logistic regression analyses were conducted to determine associations between the SLCO1B1 rs2900478 and CYP19A2 rs17601876 variants and the risk of endometrial cancer after adjusting for potential confounders, including age and gender. Multivariable logistic regression was used to assess the association between SNPs and endometrial cancer accounting for additional potential confounders, including body mass index (BMI), hypertension, diabetes, and other relevant comorbidities. The data for hemoglobin A1c (HbA1c), BMI, low-density lipoprotein (LDL), and high-density lipoprotein (HDL) were collected from the measurements closest to the time of endometrial cancer diagnosis to ensure relevance and accuracy. The selection of these covariates was based on the prior literature and their clinical relevance. We reported odds ratios (ORs) with 95% confidence intervals (CIs) to quantify the strength of the associations. The probability of endometrial cancer diagnosis-free survival was determined using the Kaplan–Meier method and with the log-rank test. All the tests were two-tailed, with *p* < 0.05 considered statistically significant.

## 5. Conclusions

The findings reported herein underscore the significance of genetic factors, particularly rs17601876, in reducing the endometrial cancer risk in the Taiwanese population. Our findings contribute to the growing understanding of the genetic architecture of endometrial cancer and highlight the potential of genetic studies for risk prediction and prevention strategies. Continued research in diverse populations is critical to uncover the global genetic landscape of endometrial cancer and to develop personalized approaches to its management.

## Figures and Tables

**Table 1:** Baseline characteristics and comorbidities of study populations.

Variables	Endometrial Cancer Group (n = 373)		Control Group (n = 3730)		*p*-Value
	n	%	n	%	
Age, years (mean/SD)	60.06 ± 10.91		60.06 ± 10.92		
Age group (mean/SD)					
<40	14 (37 ± 2.48)	3.75%	140 (37.00 ± 2.48)	3.75%	
40–60	184 (53.23 ± 5.50)	49.33%	1840 (53.23 ± 5.50)	49.33%	
>60	175 (69.09 ± 6.98)	46.92%	1750 (69.09 ± 6.98)	46.92%	
Hypertension					
No	288	77.2%	2787	74.7%	0.319
Yes	85	22.8%	943	25.3%	
Diabetes mellitus					
No	294	78.8%	3014	80.8%	0.392
Yes	79	21.2%	716	19.2%	
Chronic kidney disease					
No	323	86.6%	3196	85.7%	0.687
Yes	50	13.4%	534	14.3%	
Hyperlipidemia					
No	301	80.7%	2752	73.8%	0.004 *
Yes	72	19.3%	978	26.2%	
Body mass index (kg/m^2^)	(n = 368)		(n = 3488)		
>24	206	56.0%	1402	40.2%	<0.001 **
≤24	162	44.0%	2086	59.8%	
LDL (mg/dL), (mean/SD)	125.75 ± 43.24		105.03 ± 33.90		<0.001 **
HDL (mg/dL), (mean/SD)	54.52 ± 15.96		58.68 ± 16.45		0.001 **
Triglyceride (mg/dL), (mean/SD)	143.82 ± 85.80		110.88 ± 74.29		<0.001 **
Total cholesterol (mg/dL), (mean/SD)	195.10 ± 49.50		182.15 ± 40.92		<0.001 **
HbA1c (%), (mean/SD)	6.4 ± 1.41		5.94 ± 0.89		<0.001 **

Statistical significance is indicated by * and **, representing *p*-values of less than 0.05 and 0.001, respectively. Data are presented as mean (SD) for continuous variables while categorical variables are presented as n/t (%), where n represents the number of individuals in the category and t is the total number of individuals who provided data for that variable. HbA1c, hemoglobin A1c; BMI, body mass index; LDL, low-density lipoprotein; HDL, high-density lipoprotein.

**Table 2:** Baseline characteristics of endometrial cancer group.

Variables	Endometrial Cancer Group (n = 373)	
	n	%
Tumor size (mm)		
<20	64	17.16
20~49	118	31.64
50~99	90	24.13
>99	31	8.31
unknow	70	18.77
Clinical stage ^1^		
0~2	275	73.73
3~4	56	15.01
unknow	42	11.26
Surgery		
no	8	2.14
yes	267	71.58
unknow	98	26.27
Recurrence		
no	348	93.30
yes	25	6.70
5-year outcomes		
Survive	371	99.46
death	2	0.54
Smoking		
no	258	69.17
yes	2	0.54
Betel nut chewing		
no	260	69.71
yes	1	0.27
Alcohol consumption		
no	260	69.71
yes	1	0.27

^1^ The staging was based on 2009 FIGO staging system for endometrial cancer.

**Table 3:** Genotypes and allele frequencies of rs2900478 and rs17601876 and risk of endometrial cancer in the study population.

SNP			Case (n = 373)		Control (n = 3730)		*p*-Value
			n	%	n	%	
**rs2900478**	**non-carrier**	TT	286	76.68%	3018	80.98%	0.045 *
	**carrier**	TA/AA	87	23.32%	709	19.02%	
**rs17601876**	**non-carrier**	GG	197	52.82%	1684	45.21%	0.005 *
	**carrier**	AG/AA	176	47.18%	2041	54.79%	

Statistical significance is indicated by *, representing *p*-values of less than 0.05.

**Table 4:** Univariable logistic regression of demographics, comorbidities, and SNPs with the risk of endometrial cancer.

Variable	Univariable		
	OR	95% CI	*p*-Value
Age, years	0.998	(0.9886, 1.0084)	0.757
BMI >= 24	1.863	(1.4890, 2.3297)	<0.001 **
LDL	1.015	(1.0106, 1.0192)	<0.001 **
HDL	0.983	(0.9730, 0.9934)	<0.001 **
Triglyceride	1.004	(1.0023, 1.0052)	<0.001 **
Total cholesterol	1.007	(1.0036, 1.0101)	<0.001 **
HbA1c	1.335	(1.2011, 1.4777)	<0.001 **
rs2900478 (TA/AA)	1.196	(0.9742, 1.4694)	0.087
rs17601876 (AG/GG)	0.743	(0.6012, 0.9175)	0.006 *
Diabetes mellitus	1.081	(0.8317, 1.4051)	0.560
Chronic kidney disease	0.856	(0.6233, 1.1759)	0.338
Hyperlipidemia	0.629	(0.4801, 0.8251)	0.001 *
Hypertension	0.830	(0.6426, 1.0720)	0.154

This table presents the results of univariable logistic regression, analyzing the association between demographic factors, comorbidities, blood biomarkers, and SNPs (rs2900478 and rs17601876) with the risk of endometrial cancer. Each factor is analyzed individually without adjusting for other variables. Statistical significance is indicated by * and **, representing *p*-values of less than 0.05 and 0.001, respectively. OR, odds ratio; C.I., confidence interval; HbA1c, hemoglobin A1c; BMI, body mass index; LDL, low-density lipoprotein; HDL, high-density lipoprotein.

**Table 5:** Multivariable logistic regression of demographics, comorbidities, and SNPs with the risk of endometrial cancer.

Variable	Multivariable, Model 1			Multivariable, Model 2		
	OR	95% CI	*p*-Value	OR	95% CI	*p*-Value
Age, years	1.003	(0.9917, 1.0135)	0.648	1.002	(0.9913, 1.0132)	0.690
BMI ≥ 24	1.941	(1.5441, 2.4399)	0.000 **	1.936	(1.5399, 2.4338)	0.000 **
rs2900478 (TA/AA)	1.207	(0.9744, 1.4952)	0.085	-	-	-
rs17601876 (AG/GG)	-	-	-	0.832	(0.7067, 0.9802)	0.028 *
Diabetes mellitus	1.274	(0.9429, 1.7218)	0.115	1.281	(0.9480, 1.7316)	0.107
Chronic kidney disease	0.934	(0.6624, 1.3156)	0.694	0.950	(0.6746, 1.3370)	0.768
Hyperlipidemia	0.573	(0.4216, 0.7785)	0.000 **	0.572	(0.4212, 0.7773)	0.000 **
Hypertension	0.811	(0.5997, 1.0964)	0.173	0.806	(0.5962, 1.0898)	0.161

This model performs multivariable logistic regression to analyze the association between rs2900478 and rs17601876 and the outcome variable (having endometrial cancer or not). To reduce the impact of confounding factors, the model adjusts for demographic variables (age, BMI) and clinical conditions (diabetes mellitus, chronic kidney disease, hyperlipidemia, hypertension) minimizing potential bias. * Statistical significance is indicated by * and **, representing *p*-values of less than 0.05 and 0.001, respectively. OR, odds ratio; C.I., confidence interval; BMI, body mass index.

## Data Availability

Data is unavailable due to privacy or ethical restrictions.

## Supplementary Materials

The following supporting information can be downloaded at: https://www.mdpi.com/article/10.3390/ijms26062461/s1.
