# Supplementary material for: Genetic Variations in CYP19A1 and SLCO1B1 Genes and Their Association with Endometrial Cancer Risk in the Taiwanese Population: A Case–Control Study
Source: Int J Mol Sci. 2025 Mar 10;26(6):2461. doi: 10.3390/ijms26062461 (PMC11942030; doi:10.3390/ijms26062461)
Supplement: Supplementary file 1 [file ijms-26-02461-s001.zip › ijms-3465992-supplementary.pdf]

Supplementary Table S1. Genotype frequencies of reported genetic variants associated with endometrial cancer

	case n = 373		control n = 3730		p-value[a]
	n	%	n	%	
age	60.06	10.93	60.06	10.92	1.0000
onset age	56.41	11.03	NA	NA	NA
rs962369	23	6.2	299	8.02	0.2138
rs937213	5	1.34	95	2.55	0.1502
rs782971	219	58.71	2078	55.86	0.2898
rs7725218	234	62.73	2396	64.32	0.5421
rs7141420	281	75.34	2671	71.7	0.1364
rs4776970	141	37.9	1516	40.65	0.3025
rs4733613	0	0	0	0	NA
rs429358	59	15.82	598	16.05	0.9057
rs3820282	279	74.8	2754	73.93	0.7162
rs2900478	87	23.32	709	19.02	0.0453
rs2820292	141	37.9	1382	37.12	0.7659
rs17601876	176	47.18	2041	54.79	0.0049
rs1740828	163	43.94	1819	48.9	0.0682
rs1679014	25	6.7	292	7.83	0.4366
rs1421085	97	26.01	850	22.82	0.1639
rs1265097	32	8.58	289	7.75	0.5687
rs10938397	176	47.18	1647	44.26	0.2789

[a] Chi-square test was employed to evaluate the statistical significance of categorical variables.

Supplementary Table S2: Basic information of SNPs in SLCO1B1, including gene position, allele, SNP function annotation, and genotype frequency distribution

rsID [a]	Chr [b]	Position [c]	Ref [d]	Alt [e]	Consequence [f]	Biotype [g]	MAF [h]	TG [i]	Hom Ref [j]	Het [k]	Hom Alt [l]
**rs76096351**	12	21138543	T	C	Intron variant	Protein coding	0.014	4095	0.972	0.028	0
**rs4149018**	12	21138627	T	G	Intron variant	Protein coding	0.374	4095	0.389	0.471	0.139
**rs76610482**	12	21138859	G	T	Intron variant	Protein coding	0.042	4094	0.917	0.082	0.001
**rs571639279**	12	21141659	G	A	Splice donor variant	Protein coding	0.0001	2712	0.999	3.7E-04	0
**rs4149032**	12	21164857	T	C	Intron variant	Protein coding	0.430	4099	0.320	0.499	0.181
**rs4149033**	12	21164876	G	A	Intron variant	Protein coding	0.118	4085	0.775	0.212	0.013
**rs4149035**	12	21165331	C	T	Intron variant	Protein coding	0.119	4100	0.775	0.212	0.013
**rs10841753**	12	21168436	T	C	Intron variant	Protein coding	0.228	4099	0.599	0.344	0.057
**rs12311454**	12	21168573	G	T	Intron variant	Protein coding	0.259	4030	0.611	0.251	0.138
**rs2417957**	12	21170677	C	T	Intron variant	Protein coding	0.227	4096	0.602	0.343	0.055
**rs139257324**	12	21172734	C	T	Missense variant	Protein coding	0	4103	1	0	0
**rs373327528**	12	21172776	G	A	Missense variant	Protein coding	3.7E-04	4100	0.999	7.3E-04	0
**rs56101265**	12	21172782	T	C	Missense variant	Protein coding	0	4094	1	0	0
**rs564217518**	12	21172792	G	A	Splice donor variant	Protein coding	0	4098	1	0	0
**rs2291073**	12	21172880	T	G	Intron variant	Protein coding	0.416	4086	0.337	0.489	0.174
**rs56061388**	12	21174595	T	C	Missense variant	Protein coding	0	4102	1	0	0
**rs4149036**	12	21174806	A	C	Intron variant	Protein coding	0.470	4086	0.277	0.502	0.221
**rs2306283**	12	21176804	G	A	Synonymous variant	Protein coding	0.244	4102	0.573	0.367	0.060
**rs11045818**	12	21176827	G	A	Synonymous variant	Protein coding	1.2E-04	4089	0.999	2.5E-04	0
**rs2306282**	12	21176868	A	G	Missense variant	Protein coding	0.002	4102	0.995	0.005	0
**rs11045819**	12	21176879	C	A	Missense variant	Protein coding	1.2E-04	4101	0.999	2.4E-04	0
**rs72559745**	12	21176883	A	G	Missense variant	Protein coding	0	4096	1	0	0
**rs77271279**	12	21176898	G	T	Splice donor variant	Protein coding	0	4102	1	0	0

**rs4149049**	12	21177753	A	G	Intron variant	Protein coding	0.228	4089	0.599	0.345	0.056
**rs4149056**	12	21178615	T	C	Missense variant	Protein coding	0.110	4099	0.793	0.195	0.012
**rs4149057**	12	21178665	T	C	Synonymous variant	Protein coding	0.244	4099	0.572	0.367	0.061
**rs79135870**	12	21178957	A	G	Missense variant	Protein coding	0	4098	1	0	0
**rs11045821**	12	21179489	G	A	Intron variant	Protein coding	0.001	4099	0.998	0.002	0
**rs76551645**	12	21190554	G	A	Intron variant	Protein coding	0.019	4102	0.963	0.037	0.001
**rs11045852**	12	21196951	A	G	Missense variant	Protein coding	1.2E-04	4094	0.999	2.4E-04	0
**rs183501729**	12	21196975	C	T	Stop gained	Protein coding	0.001	4097	0.998	0.002	0
**rs11045853**	12	21196976	G	A	Missense variant	Protein coding	0	4103	1	0	0
**rs1871395**	12	21199381	A	G	Intron variant	Protein coding	0.464	4098	0.283	0.506	0.211
**rs12317268**	12	21199607	A	G	Intron variant	Protein coding	0.464	4092	0.282	0.506	0.212
**rs72559747**	12	21200544	C	G	Missense variant	Protein coding	0	4097	1	0	0
**rs59113707**	12	21202555	C	G	Missense variant	Protein coding	0	3901	1	0	0
**rs142965323**	12	21202664	G	A	Missense variant	Protein coding	0	4102	1	0	0
**rs72559748**	12	21205921	A	G	Missense variant	Protein coding	0	3842	1	0	0
**rs59502379**	12	21205999	G	C	Missense variant	Protein coding	0	4101	1	0	0
**rs4363657**	12	21215788	T	C	Intron variant	Protein coding	0.463	4079	0.282	0.505	0.214
**rs2900478**	12	21215863	T	A	Intron variant	Protein coding	0.102	4100	0.806	0.185	0.010
**rs4149071**	12	21217030	T	C	Intron variant	Protein coding	0.262	4089	0.547	0.381	0.072
**rs80208935**	12	21217107	A	G	Splice polypyrimidine tract variant/ Intron variant	Protein coding	0.012	4087	0.976	0.024	0
**rs11045872**	12	21219410	A	G	Intron variant	Protein coding	1.2E-04	4099	0.999	2.4E-04	0
**rs771622519**	12	21222361	AC	A	Frameshift variant	Protein coding	1.2E-04	4092	0.999	2.4E-04	0

**rs368052440**	12	21224839	C	T	Missense variant/ Splice region variant	Protein coding	0	4089	1	0	0
**rs4149081**	12	21225087	G	A	Intron variant	Protein coding	0.461	4088	0.282	0.510	0.208
**rs11045879**	12	21229685	T	C	Intron variant	Protein coding	0.465	4099	0.281	0.508	0.212
**rs12829704**	12	21235687	G	A	Intron variant	Protein coding	1.2E-04	4099	0.999	2.4E-04	0
**rs34671512**	12	21239042	A	C	Missense variant	Protein coding	1.2E-04	3749	0.999	2.7E-04	0
**rs55737008**	12	21239113	A	G	Missense variant	Protein coding	0	4102	1	0	0
**rs140790673**	12	21239158	C	T	Missense variant	Protein coding	0.003	4100	0.995	0.005	0

Note:

rsID [a]: Reference SNP ID

Chr [b]: Chromosome

Position [c]: Genomic position

Ref [d]: Reference allele

Alt [e]: Alternate allele

Consequence [f]: This column in genetic annotation describes the functional impact of a genetic variant (mutation) on a gene or its associated protein. It explains how the variant may affect gene expression, protein structure, or gene functionality.

Biotype [g]: This column is used to describe the type of gene, indicating whether the gene encodes a protein, produces non-coding RNA, or serves other functions.

MAF [h]: Minor allele frequency

TG [i]**:** Total genotyped

Hom Ref [j]: Homozygous reference genotype frequency

Het [k]: Heterozygous genotype frequency

Hom Alt [l]: Homozygous alternate genotype frequency

Supplementary Table S3: Basic information of SNPs in CYP19A1, including gene position, allele, SNP function annotation, and genotype frequency distribution

rsID [a]	Chr [b]	Position [c]	Ref [d]	Alt [e]	Consequence [f]	Biotype [g]	MAF [h]	TG [i]	Hom Ref [j]	Het [k]	Hom Alt [l]
rs1050787	15	51209885	A	T	3 prime UTR variant	Protein coding	0	4089	1	0	0
rs1050760	15	51210101	A	G	3 prime UTR variant	Protein coding	0	3515	1	0	0
rs4646	15	51210647	C	A	3 prime UTR variant	Protein coding	0.325	4102	0.456	0.438	0.106
rs1050677	15	51210696	G	T	3 prime UTR variant	Protein coding	0	4102	1	0	0
rs10046	15	51210789	A	G	3 prime UTR variant	Protein coding	0.468	4101	0.283	0.498	0.219
rs759973029	15	51210968	A	G	Missense variant	Protein coding	0.002	4099	0.997	0.003	0
rs78310315	15	51211010	C	T	Missense variant	Protein coding	0	4094	1	0	0
rs121434534	15	51211017	G	A	Missense variant	Protein coding	0	4100	1	0	0
rs786205108	15	51212359	TG	T	Frameshift variant	Protein coding	1.2E-04	4097	0.999	2.4E-04	0
rs2304461	15	51212359	G	A	Synonymous variant	Protein coding	1.2E-04	4097	0.999	2.4E-04	0
rs121434536	15	51212460	G	A	Missense variant	Protein coding	0	4097	1	0	0
rs80051519	15	51212489	C	T	Missense variant	Protein coding	0	4095	1	0	0
rs2289105	15	51215311	C	T	Intron variant	Protein coding	0.466	4102	0.285	0.499	0.216
rs700519	15	51215771	G	A	Missense variant	Protein coding	0.117	4103	0.780	0.205	0.015
rs786205107	15	51218539	A	G	Splice donor variant	Protein coding	0	4100	1	0	0
rs786205110	15	51218658	G	T	Splice region variant/ Splice polypyrimidine tract variant/ Intron_variant	Protein coding	0	4099	1	0	0
rs6493487	15	51221532	A	G	Intron variant	Protein coding	0.302	3871	0.461	0.438	0.101
rs121434538	15	51222349	C	T	Missense variant/ Splice region variant	Protein coding	0	3827	1	0	0
rs2899472	15	51223858	C	A	Intron variant	Protein coding	0.003	4098	0.994	0.006	0
rs1057518574	15	51227850	A	G	Missense variant	Protein coding	0	4100	1	0	0

rs774271716	15	51227853	C	CA	Frameshift variant	Protein coding	0	4101	1	0	0
rs2414095	15	51232095	G	A	Intron variant	Protein coding	0.319	4101	0.465	0.431	0.104
rs786205109	15	51236858	C	T	Splice donor variant	Protein coding	0	4094	1	0	0
rs374081925	15	51236899	G	A	Stop gained	Protein coding	0	4101	1	0	0
rs700518	15	51236915	T	C	Synonymous variant	Protein coding	0.453	4094	0.297	0.498	0.205
rs1057519118	15	51236954	C	T	Stop gained	Protein coding	0	4100	1	0	0
rs16964211	15	51238298	G	A	Intron variant	Protein coding	0.215	4089	0.616	0.338	0.046
rs7173595	15	51241539	T	C	Intron variant	Protein coding	0.322	4098	0.461	0.434	0.106
rs7175531	15	51241858	C	T	Intron variant	Protein coding	0.319	4096	0.465	0.431	0.104
rs727479	15	51242350	A	C	Intron variant	Protein coding	0.321	4095	0.462	0.433	0.105
rs2236722	15	51242798	A	G	Missense variant	Protein coding	0.047	4101	0.909	0.089	0.002
rs12907866	15	51253257	A	G	Intron variant	Protein coding	0.454	4100	0.300	0.491	0.208
rs1062033	15	51255741	C	G	Intron variant	Protein coding	0.453	4096	0.300	0.493	0.207
rs145605343	15	51260966	C	T	Intron variant	Protein coding	0.018	4099	0.965	0.035	0
rs17601876	15	51261712	G	A	Intron variant	Protein coding	0.322	4098	0.459	0.438	0.103
rs75323680	15	51269584	C	A	Intron variant	Protein coding	0.021	4091	0.958	0.042	4.9E-04
rs11636639	15	51270895	T	G	Intron variant	Protein coding	0.481	4098	0.268	0.501	0.230
rs200386728	15	51271056	TA	T	Intron variant	Protein coding	0	4040	1	0	0
rs2305707	15	51277213	A	G	Intron variant	Protein coding	0.284	4093	0.513	0.404	0.083
rs3751599	15	51281336	G	A	Intron variant	Protein coding	0.057	4101	0.889	0.108	0.003
rs7167343	15	51286397	A	G	Intron variant	Protein coding	0.146	4102	0.729	0.250	0.021
rs2470156	15	51298216	T	C	Intron variant	Protein coding	0.004	4096	0.991	0.008	2.4E-04
rs2470155	15	51298556	G	T	Intron variant	Protein coding	0.004	4099	0.991	0.008	2.4E-04
rs80056906	15	51304506	C	A	Intron variant	Protein coding	0.038	4095	0.926	0.072	0.002
rs10519302	15	51307486	A	G	Intron variant	Protein coding	0.283	4098	0.514	0.404	0.082

rs28757101	15	51307680	G	A	Intron variant	Protein coding	0.023	4102	0.955	0.044	0.001
rs192376456	15	51312586	T	C	Intron variant	Protein coding	0.024	4077	0.952	0.048	4.9E-04
rs2445762	15	51325511	T	C	Intron variant	Protein coding	0.320	4099	0.461	0.439	0.101
rs189454882	15	51330504	C	T	Intron variant	Protein coding	0.014	4098	0.972	0.027	4.9E-04
rs75484265	15	51336553	T	G	Intron variant	Protein coding	0.058	4094	0.886	0.112	0.002

Note:

rsID [a]: Reference SNP ID

Chr [b]: Chromosome

Position [c]: Genomic position

Ref [d]: Reference allele

Alt [e]: Alternate allele

Consequence [f]: This column in genetic annotation describes the functional impact of a genetic variant (mutation) on a gene or its associated protein. It explains how the variant may affect gene expression, protein structure, or gene functionality.

Biotype [g]: This column is used to describe the type of gene, indicating whether the gene encodes a protein, produces non-coding RNA, or serves other functions.

MAF [h]: Minor allele frequency

TG [i]**:** Total genotyped

Hom Ref [j]: Homozygous reference genotype frequency

Het [k]: Heterozygous genotype frequency

Hom Alt [l]: Homozygous alternate genotype frequency

Supplementary Table S4 : Stratified analysis of generalized linear model (GLM) for rs2900478 and rs17601876

	**Model 1 (rs2900478)**			**Model 2 (rs17601876)**		
Dependent variable	β	95 C.I.	*P*-value	β	95 C.I.	*P*-value
**Stratified by age**						
Reference group (31~39 years old)						
41~49 years old	-0.2341	[-1.700, -0.927]	0.311	0.03838	[-0.329, 0.405]	0.837
51~59 years old	-0.101	[-0.680, 0.229]	0.628	0.06745	[-0.269, 0.402]	0.693
61~69 years old	-0.083	[-0.496, 0.320]	0.689	0.07550	[-0.261, 0.410]	0.659
71~79 years old	-0.229	[-0.480, 0.338]	0.310	-0.04854	[-0.409, 0.310]	0.791
81~89 years old	-0.099	[-0.664, 0.224]	0.707	-0.01669	[-0.440, 0.406]	0.938
91~99 years old	-1.003	[-0.620, 0.424]	0.347	0.86304	[-0.419, 2.410]	0.215

Note:

Model 1: The model is unadjusted.

Model 2: The model is unadjusted.

Model 1 and model 2 are not adjust for the stratification variable (age) in the subgroup analysis of GLM

Abbreviation: GLM, generalized linear model; C.I., confidence interval
